# The Impact of *Lactobacillus casei* on the Composition of the Cecal Microbiota and Innate Immune System Is Strain Specific

**DOI:** 10.1371/journal.pone.0156374

**Published:** 2016-05-31

**Authors:** Busra Aktas, Travis J. De Wolfe, Nasia Safdar, Benjamin J. Darien, James L. Steele

**Affiliations:** 1 Department of Food Science, University of Wisconsin, Madison, Wisconsin, United States of America; 2 Infectious Diseases Division, Department of Medicine, University of Wisconsin, Madison, Wisconsin, United States of America; 3 William S. Middleton Veterans Affairs Hospital, Madison, Wisconsin, United States of America; 4 Animal Health and Biomedical Sciences, University of Wisconsin, Madison, Wisconsin, United States of America; University of Palermo, ITALY

## Abstract

The probiotic function to impact human health is thought to be related to their ability to alter the composition of the gut microbiota and modulate the human innate immune system. The ability to function as a probiotic is believed to be strain specific. Strains of *Lactobacillus casei* are commonly utilized as probiotics that when consumed alter the composition of the gut microbiota and modulate the host immune response. *L*. *casei* strains are known to differ significantly in gene content. The objective of this study was to investigate seven different *L*. *casei* strains for their ability to alter the murine gut microbiota and modulate the murine immune system. C57BL/6 mice were fed *L*. *casei* strains at a dose of 10^8^ CFU/day/mouse for seven days and sacrificed 3.5h after the last administration. The cecal content and the ileum tissue were collected for microbiota analysis and immune profiling, respectively. While 5 of the *L*. *casei* strains altered the gut microbiota in a strain specific manner, two of the strains did not alter the overall cecal microbiota composition. The observed changes cluster into three groups containing between 1 and 2 strains. Two strains that did not affect the gut microbiota composition cluster together with the control in their impact on pattern recognition receptors (PRRs) expression, suggesting that the ability to alter the cecal microbiota correlates with the ability to alter PRR expression. They also cluster together in their impact on the expression of intestinal antimicrobial peptides (AMPs). This result suggests that a relationship exists between the capability of a *L*. *casei* strains to alter the composition of the gut microbiota, PRR regulation, and AMP regulation.

## Introduction

Probiotics are live microorganisms, which when administered in adequate amounts, confer a health benefit on the host [1]. A diverse and rapidly expanding set of health benefits have been ascribed to probiotics including: improved ability to tolerate lactose; reduction in gastrointestinal pathogens; reduction in colorectal cancer; decrease in incidence of cold and flu; and a reduction in the symptoms associated with the inflammation-related disorders, such as ulcerative colitis [2–4]. One way to improve health of the host has been thought to be via altering the gut microbiota [5,6]. Although health benefits of probiotics are known to be strain specific [7–10], strain specificity of probiotics in their capability of modulating the composition of the gut microbiota has not been well studied.

The human gastrointestinal tract hosts over 10^14^ cells, with hundreds of different species collectively known as the microbiota [11–13]. Gut microbiota has been shown to be a major determinant in health and disease with its impact on immunity, nutrition, and pathogenesis [14]. The relationship between the complex and dynamic community of microorganisms in the gut and host immune system functions is bidirectional. This interaction is well balanced in healthy individuals and a break down can lead to gastrointestinal inflammations and metabolic disorders [15,16].

*Lactobacillus* are the most common genera from which probiotics have been derived and *L casei* is a commonly utilized probiotic species [17]. *L*. *casei* strains have been shown to alter the microbiota in the gut and influence the host immune response [18–20]. In our previous study we investigated the relationship between probiotic dose, time since probiotic consumption, changes in the gut microbiota, and immune health [20]. We have shown that *L*. *casei* 32G administration was capable of altering the murine cecal microbiota and that the alterations were dose and time dependent. We also found that the light/dark cycle has a significant impact on the composition of the cecum microbiota, hence must be taken into consideration when designing experiments that follow microbiota composition. Additionally, we demonstrated that the increase in prevalence of *Lactobacillus* in the intestinal microbiota was not directly due to the fed microorganism, *L*. *casei* 32G.

*L*. *casei* inhabits a diverse set of environmental habitats such as cheese, wine, pickle, reproductive and gastrointestinal tracts of humans and animals [21]. The population structure within the *L*. *casei* species has been analyzed by Multilocus Sequence Typing (MLST) and determined to diverge into three major lineages approximately 1.5 million years ago [22]. Subsequently, comparative genome analysis demonstrates that genome content can vary by as much as 32–45% between different strains of *L*. *casei* [23].

Since *L*. *casei* strains contain large genetic variation [23], we chose to study strain specificity of *L*. *casei*. We examined the ability of seven well characterized *L*. *casei* strains [23] to alter the composition of the gut microbiota and modulate the murine innate immune system. Additionally, we examined the relationships between pattern recognition receptors (PRRs), antimicrobial peptides (AMPs), and the gut microbiota.

## Material and Methods

### Bacterial strains

A total of seven previously described *L*. *casei* strains isolated from different ecological niches with known genome sequences were used in this study (*L*. *casei* 12A, ATCC 334, 32G, CRF28, UW-1, BL23 and M36) [23]. Stock cultures were maintained at -80°C in MRS broth (BD Difco, Sparks, MD) with 25% (v/v) glycerol (Sigma-Aldrich, St. Louis, MO). Working cultures were prepared from frozen stocks by two sequential transfers in MRS broth and incubations were conducted statically at 37°C for 24 h and 18 h, respectively. The culture was harvested by centrifugation at 5,000 rpm for 10 min at room temperature. The pellet was re-suspended in 0.85% NaCl (w/v) and the optical density at 600 nm (OD_600_) determined. A volume of washed cells (based upon the OD_600_) sufficient to yield a 25 ml cell suspension with an OD_600_ of 6.0 was harvested by centrifugation at 5,000 rpm and washed with 25 ml of 0.85% NaCl. The resulting pellet was suspended in 25 ml of 0.85% NaCl to obtain a final concentration of 10^9^ CFU/ml. The final culture solution was enumerated daily on MRS agar to confirm the dose administered to the mice.

### Animals

All procedures involving mice were conducted under the protocol #V01548 approved by the Animal Care and Use Committee of University of Wisconsin-Madison. Healthy, male C57BL/6 mice aged 8 weeks were obtained from Jackson Laboratories (Bar Harbor, ME) and group housed at University of Wisconsin-Madison Animal Health and Biomedical Science facility. Housing conditions were controlled at 25°C, 20–44% relative humidity with a 12 h light/dark cycle. Mice were fed ad libitum water and mouse chow (Harlan Teklad 7964 rodent diet, Madison, WI) throughout the study. The sample size in each group was estimated to be 6 by a sample size calculation (http://www.biomath.info/power/index.htm) with 80% power (unpaired t-test; α = 0.05) to detect a significant difference between the treatments and the control. The animals (n:48) included in this study were divided into 8 groups; each group (n:6) was administered daily 100 μl of either 0.85% NaCl (control) or one of the *L*. *casei* strains at 10^9^ CFU/ml by oral gavage for seven days. Therefore, the delivered dose was 10^8^ CFU/day/mouse.

### Sample collection

Six mice from each group were euthanized by CO_2_ asphyxiation at 3.5h after administration of the last probiotic dose. Immediately after euthanasia, the intestinal tract was removed for analysis. The cecum content was collected and the samples were immediately put on ice, and then frozen at -20°C until processed for microbial DNA extraction. Approximately 2 cm-tissue from the distal ileum was collected for RNA isolation and preserved in RNAlater (Ambion, Carlsbad, CA) overnight at 4°C. After the overnight treatment, the samples were stored at -80 °C until processing.

### DNA extraction

The cecum digesta was homogenized in 1.5ml of PBS and total DNA from 200 μl of the homogenate was isolated using the QIAamp DNA Stool Mini Kit (Qiagen Sciences, MD) with modifications to the manufacturer’s instructions. These modifications included an initial mechanical cell disruption step by inclusion of 0.1 mm glass beads (Sigma-Aldrich) followed by exposure to six 1 min beating at maximum speed in a Mini-beadbeater-96 (Biospec Products, Inc., Bartlesville, OK) with intervals of 2 min on ice. Subsequently, a heat treatment step was performed for 5 min at 95°C. The DNA was further purified by phenol:chloroform:isoamyl alcohol (25:24:1, pH 8) extraction, phase separation using Phase Lock Gels (5 PRIME) and ethanol precipitation using pellet paint co-precipitant (EMD Millipore). DNA was quantified by Qubit® 2.0 Fluorometer (Invitrogen, Carlsbad, CA). Extracted DNA was used to perform 16S rRNA sequencing.

### Ion Torrent PGM Sequencing and Microbiota Analysis

Partial 16S rRNA sequences were determined on a 318 v2 chip using the Ion Torrent Personal Genome Machine System at University of Wisconsin-Madison, Biotechnology Center. Briefly, the V1-V2 region was amplified using forward primers that contained a sample-specific bar-code with an Ion A adapter and a key sequence, while the associated reverse primer contained a truncated P1 (trP1) adapter. The sequence of these primers were: forward (8FM—5'–*CCA TCT CAT CCC TGC GTG TCT CCG AC**T CAG* BBB BBB BBB BBB BAG AGT TTG ATC MTG GCT CAG—3') with the Ion A adapter in italics, the key sequence in italics and underlined, the 13 bp bar code designated as Bs, and the 16S primer sequence in capital letters; reverse (357R - 5'–*CCT CTC TAT GGG CAG TCG GTG AT*C TGC TGC CTY CCG TA- 3') with the trP1 adapter in italics and the 16S primer sequence in capital letters. All PCR reactions were quality-controlled for amplicon saturation by gel electrophoresis. Equal quantities of each of the amplicons were pooled and purified using AxyPrep Mag PCR beads (Corning, Inc.). The resulting products were quantified using PicoGreen (Invitrogen) and Qubit fluorometer (Invitrogen) before sequencing. The data processing pipeline removed low-quality reads that: 1) did not completely match the PCR primer and barcode; 2) were shorter than 300 bp or longer than 400 bp in length; or 3) had an average quality score <22. Data analysis was performed in QIIME 1.8 framework [24]. Operational Taxonomic Units (OTUs) were generated with Uclust and chosen with QIIME picking OTU workflow based upon sequence similarity with a 97% similarity threshold. Taxonomy assignments were performed with RDP Classifier and sequences were aligned by PyNAST. Taxonomic identities were assigned using greengenes version 13_5 [25].

### RNA isolation and Gene Expression Analysis

Tissue samples from the distal small intestine were homogenized in UltraPure guanidine isothiocyanate solution (Invitrogen) using a tissue grinder with a smooth pestle (Thomas Scientific, Swedesboro, NJ). RNA was isolated using PureLink RNA mini kit (Invitrogen) as recommended by the supplier. Concentrations and purity of RNA samples were determined with a NanoDrop 2000 spectrophotometer (Thermo Scientific, Waltham, MA). Total RNA was treated with DNase I (Invitrogen) to remove DNA contamination and subsequently converted into cDNA using iScriptTM cDNA synthesis kit (Bio-Rad, Hercules, CA) according to manufacturer’s protocol. qPCR was performed using the primers shown at S2 Table and the customized 96-well prime PCR assays (Bio-rad) were used to screen 29 different genes of interest. SsoFast™ EvaGreen® Supermix (Bio-Rad) was used under the following conditions: initial denaturation at 95°C for 2 min, followed by 40 cycles of 5 sec at 95°C and 30 sec at 60°C. Data were acquired in the final step at 95°C for 5 sec and melting curves (65 to 95°C) were generated at the end for each set of primers. Gene expression was normalized to β-actin and relative gene expression was calculated by 2^-ΔΔCt^ method [26].

### Statistical analysis

For microbiota data, the statistical difference between treatments was examined using the Monte-Carlo test in package ade4 [27,28] of R 2.14.0 [29] as described by de Carcer et al [30]. The Monte Carlo test is a non-parametric test based on random permutations. The statistical differences of the between treatments was evaluated with the function of ade4::randtest.between. The values of zero were replaced with the detection limit, which is determined by the ratio of one to the lowest read number in the data set. The Benjamini-Hochberg procedure was applied to control the false discovery rate. The dominant genera that increased or decreased in abundance were identified by correspondence analysis in package ade4 of R 2.14.0 as described by de Carcer et al [30]. Statistical difference for relative gene expression was assessed with the Wilcoxon rank sum test (Mann–Whitney test) using JMP version 10 (SAS Institute Inc., Cary, NC) and was presented as mean ± SEM. Statistical difference was determined at a P value of 0.05 or less. Bacterial genera detected in cecum content of mice administered saline (control) or *L*. *casei* strains and fold change in the expression of the targeted genes in the ileum of mice administered *L*. *casei* were used to generate a dendrograms by the Ward method of hierarchical clustering (JMP version 10, SAS Institute Inc., Cary, NC).

## Results and Discussion

### Alteration of cecal microbiota by *Lactobacillus casei* administration is strain specific

Interactions between the GI microbiota and the innate immune system results in homeostasis that is critical to human health and disease [14]. Probiotics have been shown to alter the composition of the GI microbiota and is thought to be a possible mechanism by which they impact human health [6]. We previously demonstrated that *L*. *casei* 32G alters the composition of gut microbiota in mice and piglets [19,20]. Additionally we have demonstrated that *L*. *casei* strains differ 32–45% in gene content [23], hence significant strain-to-strain differences in their ability to alter the gut microbiota is likely. To evaluate the strain-to-strain differences for their ability to alter the cecal microbiota, we fed mice 1 dose (10^8^ CFU/day/mouse) daily of one of the seven previously described *L*. *casei* strains (12A, 32G, ATCC 334, BL23, CRF28, M36, and UW1). These strains were chosen based on characteristics such as ecological niche of isolation and evolutionary distance. Ion Torrent PGM sequencing of cecal content was conducted to assess the influence of *L*. *casei* strains on the murine GI microbiota. The sequencing resulted in a total of 1,546,910 filtered reads from 48 mice cecum digesta samples; the number of reads varied from 10,899 to 50,113 with an average of 32,227.3 reads per sample. To assess whether sufficient sequence reads had been collected to accurately determine the diversity of organisms present, shannon and chao1 index were examined; the results of this analysis are presented in S1 Fig. These results indicate that sufficient sequence reads were obtained to accurate describe the diversity present in these samples. After the taxonomic status of each read was assigned, 11 phyla, 20 classes, 36 orders, 61 families, 94 genera were identified.

**Fig 1:**
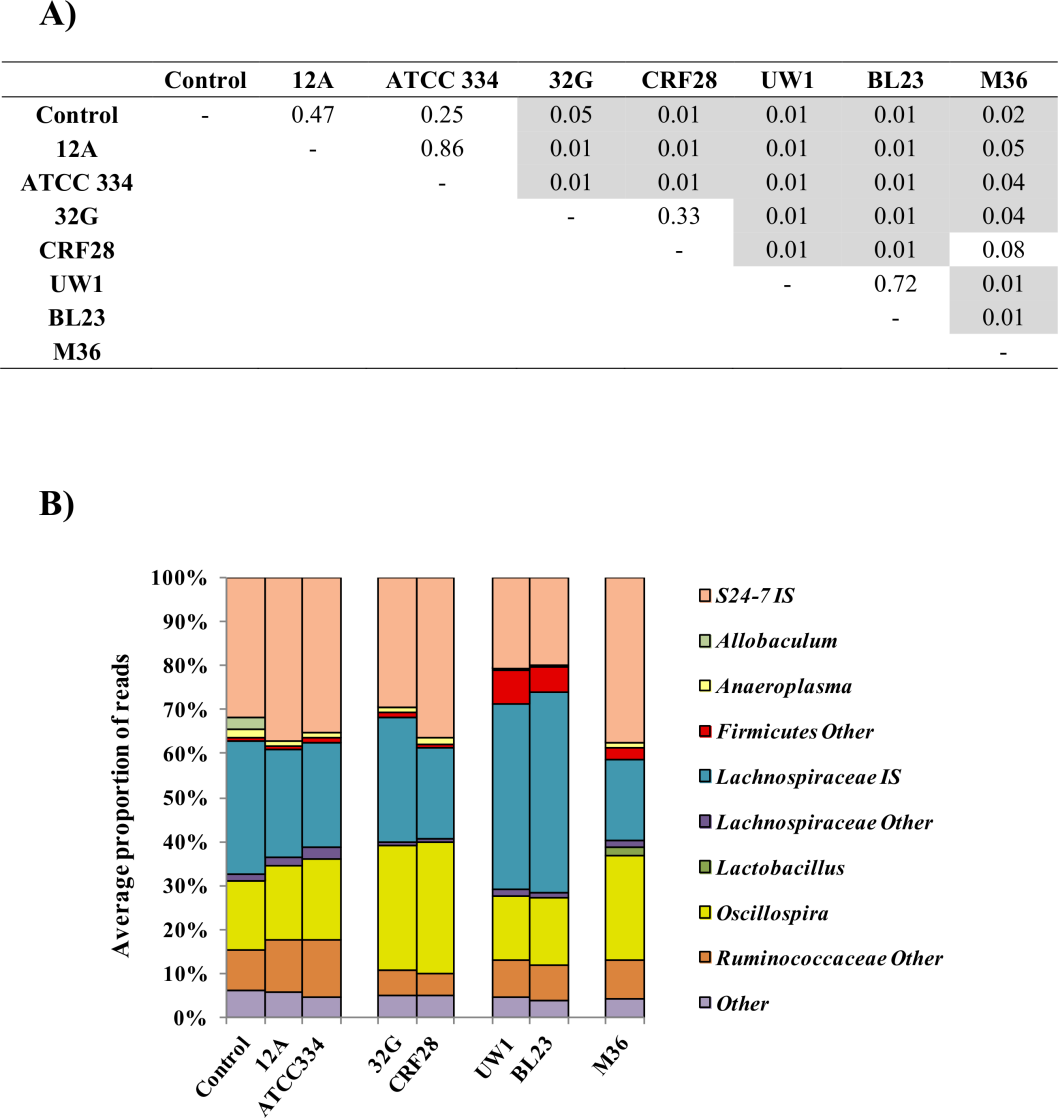
Comparison of the impact of *Lactobacillus casei* strains on the composition of cecal microbiome. Mice were administered 1 dose (10^8^ CFU/mouse/day) daily of *L*. *casei* 12A, ATCC334, 32G, CRF28, UW1, BL23 or M36 for 1 week and sacrificed 3.5h after the last dose. (A) Pair wise comparison of the OTUs at genus level detected in the cecal microbiome of each treatment. A Monte-Carlo test with 10,000 replicates was utilized to identify treatments that were significantly (p≤0.05) different. The values with p≤0.05 are highlighted. (B) Predominant genera in the cecum microbiome of mice administered *L*. *casei* strains. Treatments that are not significantly (p≤0.05) different are clustered together. Only genera that comprise greater than 5% of the total microbiome in at least one treatment are presented (n: 6 for each bar).

The cecum microbiota of the mice fed with different *L*. *casei* strains at 10^8^ CFU/day dose were evaluated at the 3.5h time point, as this was the time we previously demonstrated that the 32G bolus reached the cecum [20]. To identify treatments that were significantly (p≤0.05) different from each other a Monte-Carlo test with 10,000 replicates was utilized. Treatments that are not significantly (p≤0.05) different are clustered together. The overall OTUs detected in the cecal microbiota at the genus level for 32G, CRF28, UW1, BL23 and M36-fed mice differed significantly (p<0.05) from the control mice; while, 12A and ATCC 334-fed mice did not differ significantly from the control mice, as determined by Monte-Carlo analysis (Fig 1A). Cluster analysis and Monte-Carlo test performed with OTUs at genus level revealed that *L*. *casei* strains constitute four groups based on their influence on overall microbiota composition (Fig 1). The overall cecal microbial composition of mice fed with the strain 12A or ATCC 334 clustered with the control. The 32G and CRF28-fed mice clustered together as did UW1 and BL23; while M36 did not cluster with any of the other strains. When the samples were compared at the genus level using correspondence analysis, significant (p<0.05) changes were observed between all samples and the controls, except 12A-fed mice, with 2, 4, 6, 7, 5, and 6 changes at the genera level for ATCC 334, 32G, CRF28, UW1, BL23, and M36 respectively (Table 1). These results demonstrate that most *L*. *casei* strains are capable of altering the gut microbiota and do so in a strain specific manner.

**Table 1:** Bacterial genera detected^a^ in cecum content of mice administered saline (control) or *Lactobacillus casei* strains^b^.

Taxon	Percentage (mean ± SE)^c^^d^							
	Control	12A	ATCC334	32G	CRF28	UW1	BL23	M36
*Bacteroidales S24-7 IS*	31.8±8.6	37.0±14.7	35.2±7.3	29.3±4.9	36.3±9.2	**20.4**±**6.2**	**19.8**±**10.0**	37.6±9.5
*Lachnospiraceae IS*	30.4±9.4	24.5±6.4	24.2±7.4	28.3±10.3	**20.7**±**5.8**	**42.3**±**10.9**	**45.5**±**11.0**	**18.6**±**6.3**
*Oscillospira*	15.5±10.8	16.8±5.2	18.7±5.4	**28.5**±**11.8**	**30.0**±**7.6**	14.8±4.0	15.2±5.1	**24.1**±**5.3**
*Ruminococcaceae;Other*	9.6±4.1	11.7±3.3	13.0±6.2	**5.5**±**2.2**	**5.2**±**1.3**	8.4±1.6	8.2±1.7	8.8±2.6
*Allobaculum*	2.7±6.4	BQL	BQL	0.2±0.4	BQL	0.0±0.0	BQL	0.0±0.1
*Clostridiales;Other;Other*	2.0±0.6	1.8±0.4	**1.0**±**0.0**	2.0±0.6	1.7±0.5	2.0±0.8	2.2±0.8	**1.2**±**0.7**
*Anaeroplasma*	1.8±1.8	1.3±0.5	1.3±0.5	1.0±1.5	1.5±1.0	**0.6**±**0.5**	**0.3**±**0.5**	1.2±1.4
*Lachnospiraceae;Other*	1.7±0.5	2.2±0.8	2.3±1.5	**0.8**±**0.4**	**0.7**±**0.5**	1.5±0.3	1.5±0.5	1.5±0.8
*Clostridia;Other*	1.3±1.1	1.0±0.6	1.0±0.6	BQL	**0.2**±**0.4**	**0.4**±**0.1**	**0.3**±**0.5**	**0.4**±**0.2**
*Ruminococcus*	0.9±0.4	1.2±0.4	**1.3**±**0.5**	1.0±0.0	1.0±0.0	0.7±0.2	0.7±0.5	0.9±0.1
*Firmicutes;Other*	0.6±0.2	0.7±0.8	0.8±0.4	1.3±1.5	0.8±0.4	**7.5**±**6.7**	**5.8**±**5.7**	**2.4**±**2.7**
*Bacteria;Other*	0.4±0.1	0.2±0.4	BQL	**1.2**±**0.4**	**1.2**±**0.4**	**1.0**±**0.2**	0.2±0.4	**0.9**±**0.2**
*Ruminococcaceae IS*	0.3±0.1	BQL	0.3±0.5	0.3±0.5	BQL	**0.2**±**0.1**	BQL	0.3±0.1
*Akkermansia*	0.1±0.2	BQL	BQL	BQL	BQL	BQL	BQL	BQL
*Lactobacillus*	0.0±0.0	BQL	BQL	BQL	BQL	BQL	BQL	1.8±4.3
**Number of alterations**^**e**^	-	-	2	4	6	7	5	6

^a^Only genera that were present at ≥1% in a sample are included in this table.

^b^Mice were administered 1 dose (10^8^ CFU/mouse/day) daily of *L*. *casei* strains for 1 week and sacrificed 3.5h after the last dose.

^c^The detection limit was 0.00009 and this value was used to calculate the p-value.

^d^Genera that differ from control within each group are shown in bold (p≤0.05). The statistical difference was examined using the Monte-Carlo test.

^e^The number of genera that differed from the control for that treatment.

IS: Incertae Sedis.

BQL: Below quantifiable limit.

The overall microbiota at the phylum level differed significantly (p<0.05) from the controls in the mice fed BL23 or UW1 (S1 Table). The dominant phyla in rank order of the cecum microbiota in all of the samples were *Firmicutes* and *Bacteriodetes*. The *Firmicutes* and *Bacteroidetes* are the two major phyla found in the human and murine gut microbiota [31]. The Firmicutes/Bacteroidetes ratio has been found to have relevance to human health. For example, it has been shown that the Firmicutes/Bacteroidetes ratio was reduced in patients with Crohn’s disease, ulcerative colitis, and infectious colitis [32]. The percentage of phylum *Firmicutes* increased significantly (p < 0.05) in BL23 and UW1 groups of mice (from 65.7% to 79.5% and 78.0%) (S1 Table). These results indicate that some *L*. *casei* strains are capable of altering the gut microbiota at the phylum level.

The dominant genera in rank order of the cecum microbiota at genus level in all of the samples were *Bacteroidales S24*-7 Incertae Sedis (IS), *Lachnospiraceae* IS, and *Oscillospira*, together these genera comprise 77.5%-87% of the total microbiota (Table 1). The predominance of *S24*-7 IS decreased significantly (p < 0.05), from 31.8% to 19.8% and 20.4%, respectively, in the mice receiving the BL23 and UW1 strains. These strains were the only ones to result in a significant (p < 0.05) change in the level of *S24*-7 IS, a poorly characterized genus. The abundance of *Lachnospiraceae* IS was significantly (p < 0.05) different only in mice fed strains BL23, CRF28, M36, or UW1. While the abundance of *Lachnospiraceae* IS increased in the BL23 and UW1 group from 30.4% to 45.5% and 42.3% respectively, it decreased to 20.7% and 18.6% in the cecum of mice fed with CRF28 or M36, respectively. In contrast, Yin et al. reported a reduction in the predominance of *Lachnospiraceae* in fecal samples of BALB/c mice fed with BL23 in milk suspension compared to the mice fed with milk [33]. These differences could be due to the microbiota examined (cecum vs fecal), strain of mice examined, or administration of the bacterial culture as a milk suspension. *Lachnospiraceae* have been associated with butyrate production, which is important for epithelial cell growth [34] and has been found to be depleted in IBD patients [35]. It has been also shown that *Clostridium difficile* colonization can be controlled by a *Lachnospiraceae* isolate in germ free mice [36]. The other highly abundant genus in the cecum digesta was *Oscillospira*. Meta-analyses of human gut microbiota looking at the taxa associated with IBD have reported that *Oscillospira* abundance decreases in the subjects with Chron’s disease. Similarly, *Oscillospira* have found to be diminished in obese gut microbiota [37,38]. In our study, *Oscillospira* was significantly (p < 0.05) different only in the mice fed with 32G, CRF28 or M36 and the prevalence increased from 15.5% to 28.5%, 30% and 24.1%, respectively. Furthermore, 32G and CRF28 decreased the prevalence of *Ruminococcaceae*; *Other* from 9.6% to 5.5% and 5.2%, respectively. Predominance of genus determined as *Firmicutes*; *Other* was increased in mice cecum, after BL23, M36 and UW1 administration, from 0.6% to 5.8%, 2.4% and 7.5%, respectively. There were also marginal but significant (p<0.05) changes observed in the other genera including; *Clostridiales;Other;Other*, *Anaeroplasma*, *Lachnospiraceae;Other*, *Clostridia;Other*, *Ruminococcus*, *Bacteria;Other*, and *Ruminococcaceae* IS, which are presented in Table 1. Although we fed the mice with *L*. *casei* strains, the *Lactobacillus* genus was not one of the predominant bacterial genera detected in the cecum. This result suggests that the abundance of *Lactobacillus* was lower than the detection limit of Ion Torrent PGM sequencing. In our previous study, we demonstrated that the lactobacilli that increased in prevalence in the cecum microbiota of the mice fed *L*. *casei* 32G was not *L*. *casei*, rather it was *L*. *johnsonii*, a commensal lactobacilli present in the murine gut [39,40]. These results indicate that some strains are capable of altering the gut microbiota at the genus level. The observed changes cluster into three groups containing between 1 and 2 strains. The ability to alter the composition of the gut microbiota is likely to influence health, as numerous publications indicate that the gut microbiota impacts the health of the host.

### Effect of *Lactobacillus casei* strain on intestinal barrier and innate immune system is strain specific

**Fig 2:**
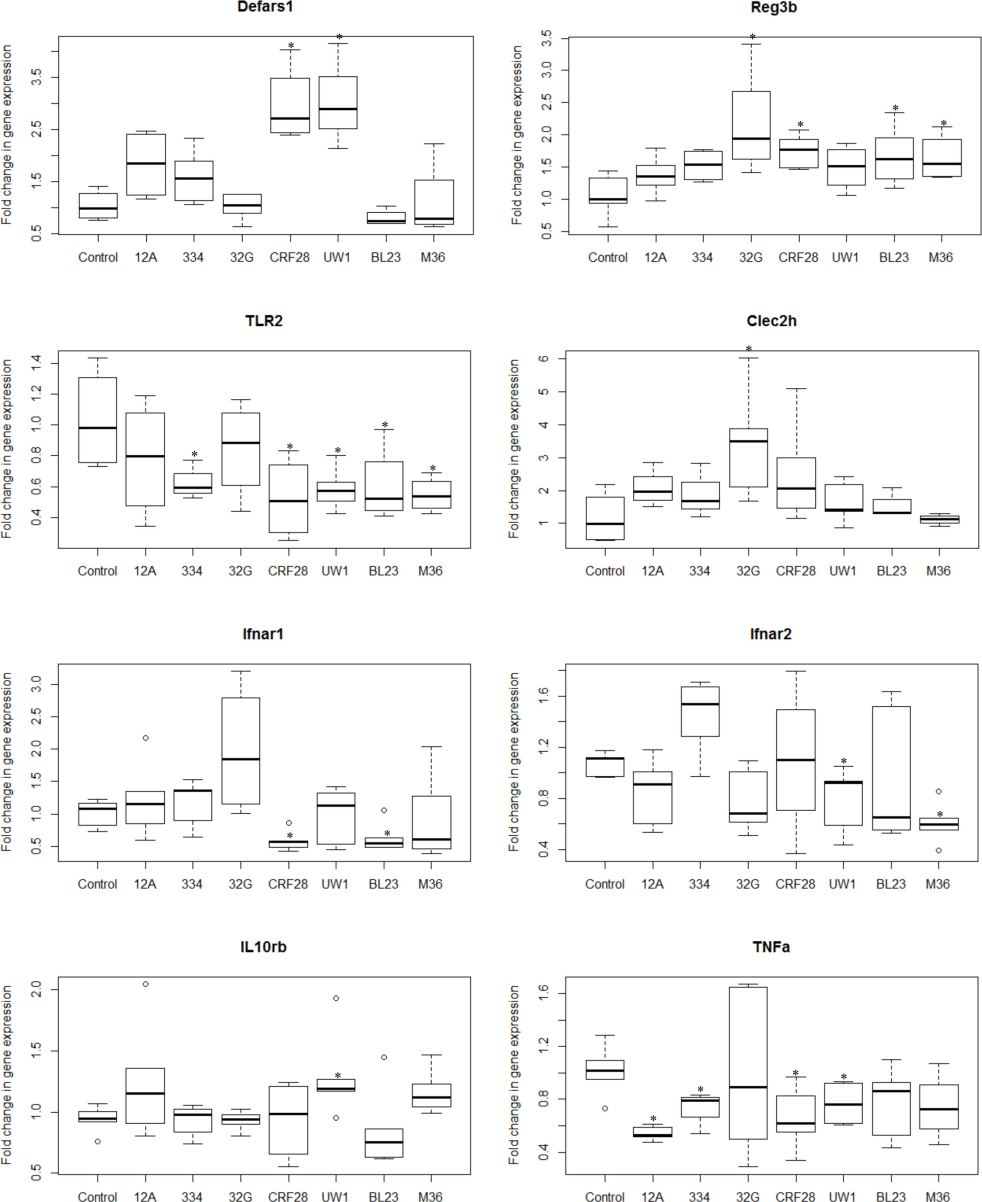
Fold change in gene expression of antimicrobials, pattern recognition receptors, and cytokines in the ileum of mice administered *L*. *casei* 12A, ATCC 334, 32G, CRF28, UW-1, BL23 or M36. The strains were administered 1 dose (10^8^ CFU/ mouse) daily for 1 week and sacrificed 3.5h after the last dose; * p<0.05: significant differences from the control, (n: 6/group).

There is a well-balanced relationship between microbiota and the immune system in healthy individuals. A disruption in this balance can lead to gastrointestinal inflammation and metabolic disorders [16]. Probiotics have been shown to alter the composition of the gut microbiota and the expression of genes involved in host innate immunity [41,42]. In this study, we have demonstrated that *L*. *casei* strains alter the gut microbiota in a strain specific manner. Since there is a bidirectional interaction between the gut microbiota and host immunity, variation in the immunomodulatory capacity of individual *L*. *casei* strains is also likely. To evaluate the effect of different strains on mice intestinal barrier and innate immunity, we isolated total RNA from ileal tissue to establish a gene expression profile in mice subjected to one of the *L*. *casei* strains. We targeted 26 genes associated with the innate immune functioning and intestinal barrier. Expression of *IL-12p35* and *IL-12p40* genes were below the limit of detection in all samples evaluated. Eleven of the targeted genes (*Lyz2*, *Defa3*, *Defa20*, *Defa21*, *Defa22*, *Defa23*, *Defa24*, *Defa-rs7*, *Pigr*, *ZO-1*, *IL-10ra*,) showed no statistical differences from the control in all samples evaluated (data not shown). Thirteen of the targeted genes (*Defa-rs1*, *Lyz1*, *Reg3β*, *Reg3γ*, *Clec2h*, *TLR2*, *TLR4 Ifnar1*, *Ifnar2*, *IL-10rb*, *Tnf-α*, *Occludin*, and *ZO-2*) were expressed statistically (p<0.05) different that the expression in control mice in some samples and these results are presented in Fig 2 and S2 Fig.

**Fig 3:**
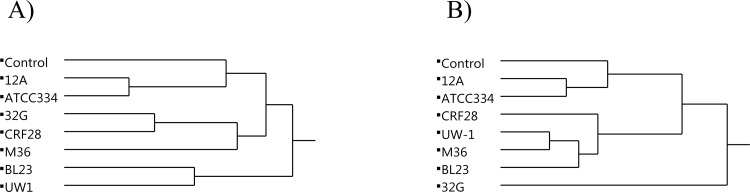
**Hierarchical clustering of seven *Lactobacillus casei* strains and the control based on their impact on mouse cecal microbiota (A) and pattern recognition receptors in the murine ileum (B).** The results presented in average.

Intestinal cells recognize microbial ligands via pattern recognition receptors (PRRs) that contribute to the interaction between the gut microbiota and the innate immune system [43]. We screened intestinal PRRs including Toll-like receptors (TLRs) and C-type lectin like receptor 2h (Clec2h) to evaluate the effect of different *L*. *casei* strains on the expression of PRRs. TLRs induce the secretion of inflammatory cytokines, are involved in the maintenance of tight junctions between intestinal epithelial cells and the production of AMPs [44]. TLR4 is required for recognition of Gram (-) bacteria whereas TLR2 is the main receptor that recognizes Gram (+) bacteria [45]. TLR2 expression was significantly (p<0.05) decreased in mice fed strains ATCC 334, CRF28, UW1, BL23, and M36 whereas TLR4 expression was decreased only in mice fed BL23 and M36 (Fig 2 and S2 Fig). However, strains 12A and 32G did not have any significant effect on TLR expression. These results suggest that alteration in the expression of TLRs is a strain specific trait. The strains might have an indirect mechanism of action in suppressing the expression of TLRs. For example, *L*. *casei* strains might compete with commensals that stimulate expression of TLRs, thereby reducing TLR gene expression [46–48]. Another PRR we examined, Clec2h, was significantly (p < 0.05) up-regulated only in the mice fed-32G. A significant increase in Clec2h expression was also observed in our previous study with 32G fed mice. The function of Clec2h is poorly defined; however, it is thought to have a role in regulating innate immune responses [49]. These results demonstrate that administration of some of the *L*. *casei* strains modify the expression of TLRs and strain 32G consistently up-regulates the Clec2h expression. Additionally, cluster analysis of overall changes in expression of PRRs examined in this study revealed that ATCC 334 and 12A cluster together with the control; the clustering of the control with ATCC 334 and 12A also occurred in the composition of the cecal microbiota analysis (Fig 3). These results indicate that the ability of a strain to alter the cecal microbiota correlates with the ability to alter PRR expression.

AMPs are crucial components of innate immune system as an active intestinal mucosal defense and have an important role in shaping the composition of the intestinal microbiota [50–52]. Some AMPs, such as REG3g, require signals from commensal bacteria to be expressed, while others like lysozymes and some defensins are expressed independent of bacterial signals [53–55]. The bidirectional relationship between defensins and gut microbiota has been supported in previous studies. Menendez et al. reported that the host microbiota regulated ileal alpha defensin expression in mice exposed to oral antibiotic administration [56]. In addition, paneth cells alpha defensins have been shown to be essential in homeostatic control and shaping of the composition of intestinal microbiota [57]. In this study we examined the expression of different AMPs in the mouse small intestine to evaluate the influence of different *L*. *casei* strains on the expression of genes encoding AMPs. Strains 12A and ATCC 334 had no significant effect on the expression of AMPs in the murine small intestine (Fig 2). The other *L*. *casei* strains examined in this study varied in their effect on AMP gene expression (Fig 2). Administration of CRF28 and UW1 resulted in a significant (p < 0.05) increase in Defa-rs1 expression compared to the control mice. The expression of Reg3b was increased by strains 32G, CRF28, BL23 and M36. Lysozyme expression was decreased significantly (p < 0.05) only by M36. Expression of Reg3g was increased significantly (p < 0.05) only by strain 32G (S2 Fig). *L*. *casei* ATCC 334 and 12A were the only strains examined which did not alter the expression of any of the AMPs examined in this study (Table 2). Additionally, these two strains were the only ones that did not alter the murine gut microbiota. The other five strains alter the expression of the AMPs in a strain specific manner. Remarkably, even though we examined only a subset of the intestinal AMPs, the other 5 strains could be differentiated from each other based solely upon their influence on the expression of Defa-rs1, Reg3β, Reg3g, and lysozyme (Table 2). These results suggest a relationship exist between *L*. *casei* strains, AMP regulation, and cecal microbial composition.

**Table 2:** Change in gene expression of AMPs of mouse ileum fed with *Lactobacillus casei* strains.

	ATCC 334	12A	32G	CRF28	UW-1	BL23	M36
**Defa-rs1**	NSC	NSC	NSC	↑	↑	NSC	NSC
**Reg3β**	NSC	NSC	↑	↑	NSC	↑	↑
**Reg3g**	NSC	NSC	↑	NSC	NSC	NSC	NSC
**Lysozyme**	NSC	NSC	NSC	NSC	NSC	NSC	↓

↑: significant increase in the gene expression. ↓: significant decrease in the gene expression. NSC: No significant change in the gene expression.

PRRs regulate cytokine activity in response to microbial surface patterns. Cytokines are critical in the regulation and development of the innate immune system [58]. The effect of *L*. *casei* strains on both inflammatory and anti-inflammatory cytokines was analyzed and the strain-to-strain differences evaluated in this study. A significant (P<0.05) decrease in expression of TNF-α, a pro-inflammatory cytokine, was observed in mice fed-12A, ATCC 334, CRF28 and UW1, relative to the control mice (Fig 2). Interferons (IFN) are another group of inflammatory cytokines which signal via interferon receptors (IFNar) [59]. A reduction in the expression of the interferon receptor 1 (IFNar1) in mice fed-CRF28 and BL23 was observed. Additionally, a reduction in the expression of Interferon receptor 2 (IFNar2) was observed in mice fed-UW1 and M36. These results are significant as interferon receptors (IFNar) are required for IFNs to mediate inflammatory responses. Additionally, expression of Il-10rb, an anti-inflammatory marker, was significantly (p<0.05) increased by UW1 (Fig 2). IL-10r is required to activate members of the IL-10 subfamily of cytokines and a deficiency in IL-10rb leads to inflammatory bowel disease [60]. IL-10 suppresses the production of pro-inflammatory cytokines such as TNF-α and regulates inflammatory responses in the host [61]. Alternatively, the reduction in TNF-α expression could be due to the reduction in TLR2 expression, as TLR2 has been demonstrated to induce the production of inflammatory cytokines like TNF-α [62,63]. These results suggest that administration of *L*. *casei* strains tends to have an anti-inflammatory effect on the murine immune system that the extent and mechanism are strain specific.

Interactions between the host intestinal epithelial barrier and the gut commensal microbiota has a significant role in the health of the host in the gut [64,65]. Host intestinal epithelial barrier function is dependent on both the level and the distribution of tight junction proteins (TJPs) [66,67]. To evaluate the impact of different *L*. *casei* strains on TJPs production, we examined ileal expression of Occludin and Zonula Occludens (ZO) after administration of *L*. *casei* strains. We observed a significant (p < 0.05) increase in Occludin expression in mice fed-ATCC 334, CRF28, UW1, BL23 and M36 and a marginal increase in expression of ZO-2 noted in mice fed-12A and BL23 (S2 Fig). These results suggest that most *L*. *casei* strains are capable of strengthening intestinal epithelial barrier function via an increase in TJP gene expression.

## Conclusion

**Fig 4:**
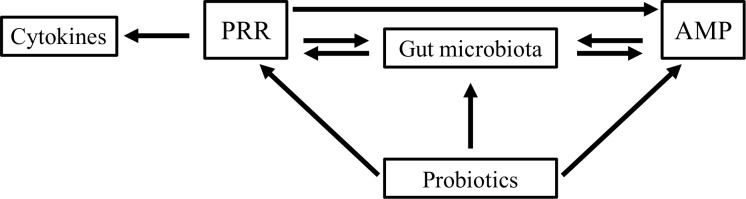
Proposed interaction between probiotics and the gut microbiota. PRR, pattern recognition receptor; AMP, antimicrobial peptide.

Variation in *L*. *casei* gene content is known to be large [23]; therefore, we chose to examine *L*. *casei* strain-to-strain variation in the ability to alter the gut microbiota and modulate the host immune system. This study has demonstrated that large strain-to-strain variation does exist with the *L*. *casei* species with regard to their ability to modulate the host gut microbiota and the host immune system. Additionally, our results indicate that there is a relationship between a strain’s ability to alter the composition of the gut microbiota, PRR regulation, and AMP regulation (Fig 4).

## Supporting Information

S1 Fig Alpha rarefaction plots based on Chao1 (A) and Shannon index (B).
(PDF)

S2 Fig Fold change in gene expression of antimicrobials, tight junction proteins, and pattern recognition receptor in the ileum of mice administered *L*. *casei* 12A, ATCC 334, 32G, CRF28, UW-1, BL23 or M36. The strains were administered 1 dose (10^8^ CFU/ mouse) daily for 1 week and sacrificed 3.5h after the last dose; * p<0.05: significant differences from the control, (n: 6/group).
(PDF)

S1 Table Bacterial phyla detected in cecum content of mice administered saline (control) or *Lactobacillus casei* strains. (PDF)

S2 Table Primers used in qPCR analysis. (PDF)
