# Supplementary figures and images for: The Impact of Lactobacillus casei on the Composition of the Cecal Microbiota and Innate Immune System Is Strain Specific
Source: PLoS One. 2016 May 31;11(5):e0156374. doi: 10.1371/journal.pone.0156374 (PMC4887021; doi:10.1371/journal.pone.0156374)

A)

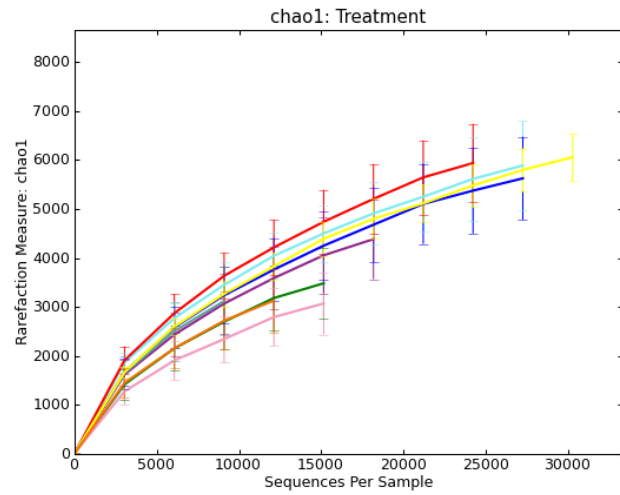

B)

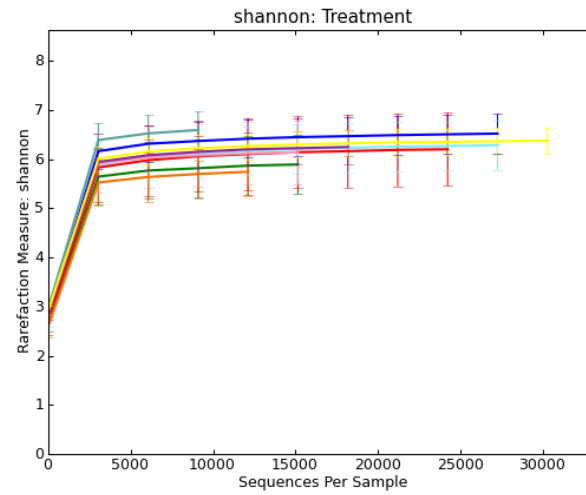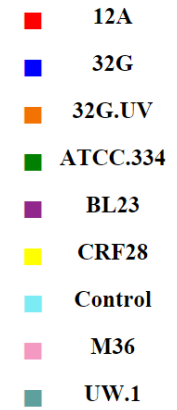

**Figure S1.** Alpha rarefaction plots based on Chao1 (A) and Shannon index (B).

Supplement: S1 Fig — Alpha rarefaction plots based on Chao1 (A) and Shannon index (B). (PDF) [file pone.0156374.s001.pdf]
