# Supplementary material for: The Impact of Lactobacillus casei on the Composition of the Cecal Microbiota and Innate Immune System Is Strain Specific
Source: PLoS One. 2016 May 31;11(5):e0156374. doi: 10.1371/journal.pone.0156374 (PMC4887021; doi:10.1371/journal.pone.0156374)
Supplement: S2 Fig — The strains were administered 1 dose (108 CFU/ mouse) daily for 1 week and sacrificed 3.5h after the last dose; * p<0.05: significant differences from the control, (n: 6/group). (PDF) [file pone.0156374.s002.pdf]

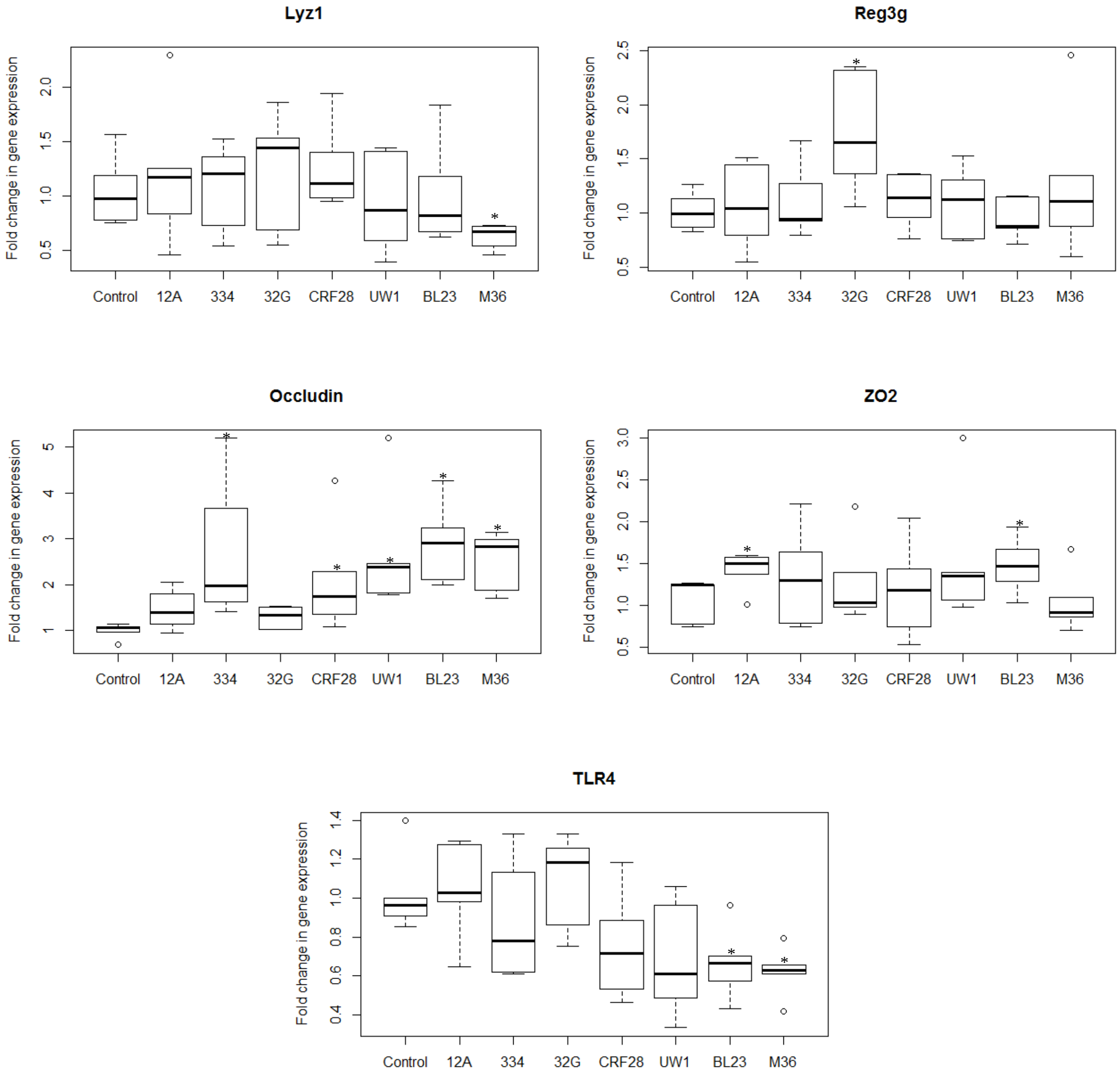

Figure S2. Fold change in gene expression of antimicrobials, tight junction proteins, and pattern recognition receptor in the ileum of mice administered *L. casei* 12A, ATCC 334, 32G, CRF28, UW-1, BL23 or M36. The strains were administered 1 dose (10^8 CFU/ mouse) daily for 1 week and sacrificed 3.5h after the last dose; * p<0.05: significant differences from the control, (n: 6/group).
