# Supplementary material for: The Impact of Lactobacillus casei on the Composition of the Cecal Microbiota and Innate Immune System Is Strain Specific
Source: PLoS One. 2016 May 31;11(5):e0156374. doi: 10.1371/journal.pone.0156374 (PMC4887021; doi:10.1371/journal.pone.0156374)
Supplement: S1 Table — (PDF) [file pone.0156374.s003.pdf]

Table S1. Bacterial phyla detected in cecum content of mice administered saline (control) or *Lactobacillus casei* strains[b].

Taxon	Percentage (mean ± SE)[bc]							
	Control	12A	32G	ATCC334	BL23	CRF28	M36	UW1
Bacteroidetes	31.8 ± 8.5	37.2 ± 14.7	29.2 ± 4.8	35.2 ± 7.4	**19.8 ± 10.1**	36.5 ± 9.1	37.6 ± 9.4	**20.5 ± 6.2**
Firmicutes	65.7 ± 8.2	61.0 ± 14.4	68.7 ± 4.0	63.2 ± 7.2	**79.5 ± 10.1**	61.0 ± 9.6	60.2 ± 9.6	**78.0 ± 6.1**
Tenericutes	1.8 ± 1.8	1.5 ± 0.6	1.0 ± 1.4	1.3 ± 0.6	**0.3 ± 0.3**	1.5 ± 1.0	1.2 ± 1.4	**0.6 ± 0.4**
Proteobacteria	0.1 ± 0.3	0.0 ± 0.0	0.0 ± 0.0	0.0 ± 0.0	0.0 ± 0.0	0.0 ± 0.0	0.0 ± 0.0	0.0 ± 0.0
Verrucomicrobia	0.1 ± 0.2	0.0 ± 0.0	0.0 ± 0.0	0.0 ± 0.0	BQL	0.0 ± 0.0	0.0 ± 0.0	0.0 ± 0.0
Bacteria;Other	0.4 ± 0.2	0.4 ± 0.1	**1.0 ± 0.3**	**0.2 ± 0.1**	0.4 ± 0.1	**1.0 ± 0.4**	**0.9 ± 0.2**	**1.0 ± 0.2**
Actinobacteria	0.0 ± 0.0	0.0 ± 0.0	0.0 ± 0.0	0.0 ± 0.0	0.0 ± 0.0	0.0 ± 0.0	0.0 ± 0.0	0.0 ± 0.0
Deferribacteres	0.0 ± 0.0	0.0 ± 0.0	0.0 ± 0.0	BQL	0.0 ± 0.0	BQL	0.0 ± 0.0	BQL
Cyanobacteria	0.0 ± 0.0	0.0 ± 0.0	BQL	BQL	BQL	BQL	0.0 ± 0.0	BQL
TM7	0.0 ± 0.0	BQL	0.0 ± 0.0	BQL	BQL	BQL	BQL	BQL
Thermi	BQL	BQL	0.0 ± 0.0	BQL	0.0 ± 0.0	BQL	BQL	0.0 ± 0.0
**Number of alterations**[d]	-	-	1	1	3	1	1	4

[a]The detection limit was 0.00009 and this value was used to calculate the p-value.
[b]Mice were administered 1 dose (10^8 CFU/mouse/day) daily of *L. casei* strains for 1 week and sacrificed 3.5h after the last dose.
[c]Phyla that differ from control within each group are shown in bold ($p \leq 0.05$).
[d]The number of genera that differed from the control for that treatment.
IS: Incertae Sedis.
BQL: Below quantifiable limit.
