# Supplementary material for: The Impact of Lactobacillus casei on the Composition of the Cecal Microbiota and Innate Immune System Is Strain Specific
Source: PLoS One. 2016 May 31;11(5):e0156374. doi: 10.1371/journal.pone.0156374 (PMC4887021; doi:10.1371/journal.pone.0156374)
Supplement: S2 Table — (PDF) [file pone.0156374.s004.pdf]

Table S2. Primers used in qPCR analysis

Gene	Forward	Reverse	Reference
Occludin	5'-CCCTGACCACTATGAAACAG-3'	5'-TTGATCTGAAGTGATAGGTG-3'	Corridoni et al., 2012
ZO-1	5'-CCTAAGACCTGTAACCATCT-3'	5'-CTGATAGA- TATCTGGCTCCT-3'	Corridoni et al., 2012
ZO-2	5'-CTAGACCCCCAGAGCCCCAGAAA-3'	5'-TCGCAGGAGTCCACGCATACAAG-3'	Ukena et al., 2007
